# Strength of patient cohorts and biobanks for cardiomyopathy research

**DOI:** 10.1007/s12471-020-01456-4

**Published:** 2020-08-11

**Authors:** R. A. de Boer, L. L. A. M. Nijenkamp, H. H. W. Silljé, T. R. Eijgenraam, R. Parbhudayal, B. van Driel, R. Huurman, M. Michels, J. Pei, M. Harakalova, F. H. M. van Lint, M. Jansen, A. F. Baas, F. W. Asselbergs, J. P. van Tintelen, B. J. J. M. Brundel, L. M. Dorsch, M. Schuldt, D. W. D. Kuster, J. van der Velden, DOSIS consortium

**Affiliations:** 1 Department of Cardiology, University Medical Center Groningen, University of Groningen, Groningen, The Netherlands; 2 Department of Cardiology, University Medical Center Rotterdam, Erasmus MC, Rotterdam, The Netherlands; 3 Department of Cardiology, Division Heart & Lungs, University Medical Centre Utrecht, Utrecht University, Utrecht, The Netherlands; 4 Department of Genetics, University Medical Centre Utrecht, Utrecht University, Utrecht, The Netherlands; 5 Department of Physiology, Amsterdam Cardiovascular Sciences, Amsterdam UMC, Vrije Universiteit, Amsterdam, The Netherlands

**Keywords:** Cardiomyopathies, Gene variants, Patient cohorts

## Abstract

In 2011 the Netherlands Heart Foundation allocated funding (CVON, Cardiovasculair Onderzoek Nederland) to stimulate collaboration between clinical and preclinical researchers on specific areas of research. One of those areas involves genetic heart diseases, which are frequently caused by pathogenic variants in genes that encode sarcomere proteins. In 2014, the DOSIS (**D**eterminants **o**f **s**usceptibility **i**n inherited cardiomyopathy: towards novel therapeutic approache**s**) consortium was initiated, focusing their research on secondary disease hits involved in the onset and progression of cardiomyopathies. Here we highlight several recent observations from our consortium and collaborators which may ultimately be relevant for clinical practice.

## Dutch contribution to the field

- DOSIS represents a national research consortium on cardiomyopathies.
- DOSIS researchers have shown more severe diastolic dysfunction in female than male HCM patients at the time of surgery.
- Indexation for body size is needed to set the diagnostic threshold for left ventricular thickening in HCM.
- Cell-to-cell variability is present in the hearts of patients with a Dutch founder mutation.

## Introduction

**Fig. 1:**
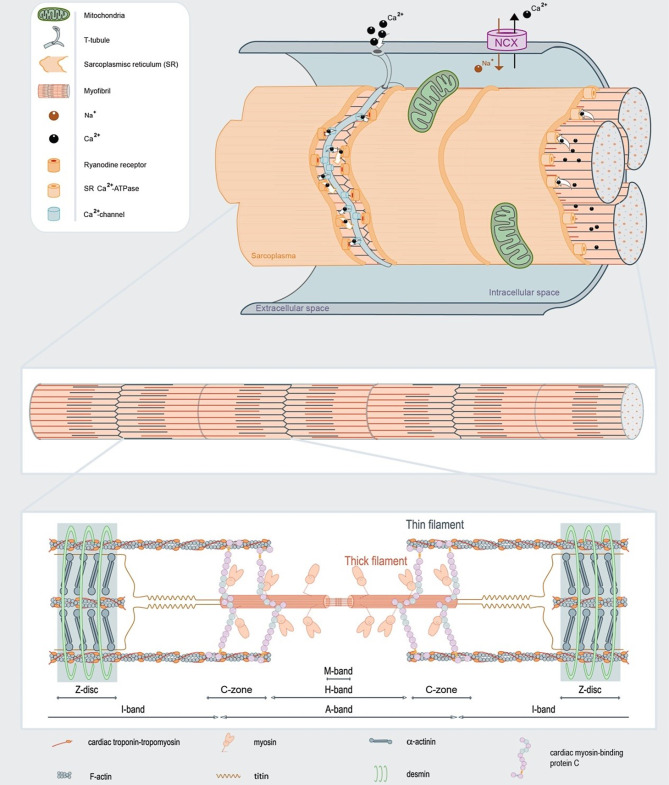
Activation of a cardiomyocyte triggers calcium entry and release of calcium from the sarcomplasmic reticulum (*SR*), which results in contraction of the cardiac myofibrils. To relax the cardiomyocyte, calcium is pumped back into the SR, and out of the cell via the sodium-calcium exchanger (*NCX*). Myofibrils consist of sarcomeres composed of the thin actin and thick myosin filament, and the third filament titin. Sarcomeres consist of sub-regions (depicted by the different bands), which underlie the striated pattern of cardiac muscle. Gene variants that cause cardiomyopathies are frequently found in myosin heavy chain, troponin T, cardiac myosin-binding protein‑C (located in the Z‑zone) and titin. (Figure is adapted from Sequeira at al. [34])

Inherited cardiomyopathies, caused by pathogenic variants in genes encoding proteins that regulate cardiomyocyte contractility, are a major cause of morbidity and mortality. In 50–60% of familial hypertrophic cardiomyopathy (HCM) and 30–40% of dilated cardiomyopathy (DCM), a pathogenic gene variant can be identified. The most common genes that are affected in HCM are *MYH7, MYBPC3 *and *TNNT2, *which encode the thick filament proteins myosin heavy chain, cardiac myosin-binding protein‑C (cMyBP-C), and the thin filament protein troponin T. In DCM the titin (*TTN*) gene, which encodes the giant myofilament protein titin, is the most frequently affected gene (~15–20% of all gene variants) [1]; in particular gene variants that lead to *TTN* truncation have been shown to be pathogenic [2]. In addition, in the Netherlands, a founder mutation in the *PLN *gene, which encodes the calcium-handling protein phospholamban, was identified in 2012 as a now well-known cause of DCM and arrhythmogenic cardiomyopathy [3, 4]. Fig. 1 depicts a cardiomyocyte to illustrate the affected proteins involved in cardiomyopathies. Upon activation of a cardiomyocyte, calcium enters the cell via the L‑type calcium channel, which subsequently releases calcium from the intracellular calcium store, the sarcoplasmic reticulum. Calcium binds to the troponin complex, which induces a conformational change of troponin-tropomyosin, and thereby releases binding sites for myosin heads on the thin actin filament. Binding of myosin to actin (so-called cross-bridge) results in force development. The kinetics of cross-bridge cycling is regulated by cMyBP‑C, and the giant protein titin, encoded by *TTN* and linked with DCM, underlies passive stiffness of sarcomeres [5].

In past years the number of genes in genetic diagnostic panels has increased with the hope to identify a disease-causing variant in a larger group of patients and their family members. However, recent studies conclude that the additional benefit of screening large numbers of genes is disappointingly low and of marginal clinical utility [6, 7]. Numerous new *TTN *gene variants have been identified, mainly because of its large size. A study in three European cardiogenic centres showed that missense and non-frameshifting insertions/deletions variants are most likely benign, as reference populations showed comparable frequencies of these rare *TTN *variants [8]. The current panels thus rather increase the number of variants with unknown significance, which are likely benign, though they may have a modifier role in disease. Cardiomyopathy patients with a suspected genetic aetiology should be referred for genetic screening. For HCM this includes patients with asymmetric left ventricular hypertrophy, not explained by abnormal loading conditions, with or without a clear family history. For DCM this includes patients with non-ischaemic DCM, not fully explained by other aetiological factors. Young age of onset and familial occurrence are important parameters that hint towards a genetic aetiology. However, late onset in seemingly sporadic cases does not exclude a genetic origin due to the reduced penetrance and variable disease expression. All HCM and DCM patients in whom genetic screening was performed can be added to the biobanks, including their relatives (asymptomatic mutation carriers).

**Fig. 2:**
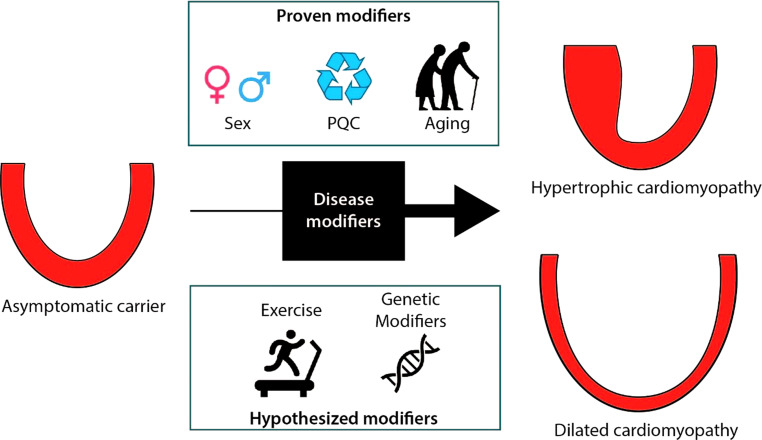
Disease modifiers in inherited cardiomyopathies (*PQC* protein quality control)

Since cardiomyopathies continue to constitute one of the most common causes of sudden cardiac death in the young and still represent major causes for cardiac transplantation, adequate identification of additional disease triggers and understanding the pathomechanisms is of utmost importance. The clinical approach is furthermore complicated since inherited cardiomyopathies are clinically heterogeneous: age-dependent penetrance and disease-severity differ greatly between patients with the identical genetic variant. The mechanisms that underlie the variation in disease expression are still largely unknown. By combining cellular, genetic and clinical data from well-phenotyped national patient cohorts, DOSIS strived to define disease factors (i.e. secondary hits) that in addition to the pathogenic gene variant cause and aggravate cardiac disease in cardiomyopathy patients (Fig. 2; Tab. 1). Several recent observations are highlighted below.

**Table 1:** Cohorts of DOSIS

Cohort	Participating centres	Population	Biobank collection	Aim
Erasmus HCM observational cohort	Erasmus MC	HCM patients and gene variant carriers	DNA	Identify predictive clinical markers for major cardiac events
BIO FOr CARe observational cohort	UMC Utrecht, UMC Groningen, Amsterdam UMC, Erasmus MC	All sarcomere gene variants	DNA, RNA (from blood), plasma and serum	Identify predictive biomarkers for major cardiac events
Telephone interviews	UMC Utrecht, UMC Groningen, Amsterdam UMC	*MYBPC3* founder gene variant carriers	n.a.	Determine predictive value of environmental factors (especially exercise) for major cardiac events
ENERGY randomised placebo-controlled trial	Amsterdam UMC, Erasmus MC	Preclinical *MYH7* gene variant carriers	Serum	Determine effects of trimetazidine on improving myocardial energy efficiency in the pre-clinical disease stage
Myectomy cohort	Erasmus MC, UMC Utrecht	HCM patients undergoing septal myectomy	DNA, cardiac tissue	Collect myocardial tissue for use in etiological studies

## Indexation for body size to set the diagnostic threshold for left ventricular thickening

Using a large collection of myectomy samples from patients with obstructive HCM, we have shown that there is a sex-specific difference in diastolic function at the time of myectomy in HCM patients carrying pathogenic variants in *MYH7* and *MYBPC3* [9]. Women showed more diastolic dysfunction evident from significantly higher E/e’ ratios, impaired left ventricular filling patterns, and higher tricuspid regurgitation velocities. Of the female patients, 50% showed grade III diastolic dysfunction, while the majority of male patients (56%) had only mild (grade I) diastolic dysfunction. Correction of maximal septal thickness and left atrial diameter for body surface area (BSA) resulted in significantly higher values in female compared with male patients. Histological and protein analyses revealed more advanced remodelling of the heart in female compared with male HCM patients evident from higher levels of fibrosis and activation of the cardiac foetal gene program, which is characteristic of heart failure. In addition to genetic screening, the current diagnostic criterion of hypertrophy is a maximal left ventricular wall thickness of ≥15 mm, or ≥13 mm in first-degree relatives of HCM patients. As the hearts of women, and in general relatively small persons, are smaller than the hearts of men, this threshold for the diagnosis of HCM should probably be corrected for body size [10]. The current diagnostic threshold, which does not take into account body size, may likely explain the male predominance in HCM patient cohorts, simply because males in general have larger hearts. A recent study in a Dutch cohort of 199 genotype-positive subjects, family members of HCM patients who were referred for cardiac screening between 1995 and 2018, indexation of wall thickness by BSA decreased the number of HCM diagnoses [11]. Moreover, predictive accuracy for HCM-related events (mortality, cardiac transplantation, implantable cardioverter-defibrillator implantation and septal reduction therapy) improved significantly after indexation by BSA. These studies indicate that correcting left ventricular thickness for body size should be considered for the diagnosis of HCM and longitudinal follow-up studies in larger cohorts of preclinical genotype-positive individuals are needed to confirm this.

## Altered metabolism as key driver of disease in cardiomyopathies

Several studies suggest an important role for secondary disease modifiers such as additional epigenetic and genetic variations and environmental disease triggers. Compelling data have accumulated that obesity is an overarching risk factor, also for age-of-onset and severity of cardiomyopathies. Proof that obesity contributes to disease onset and severity comes from cohort studies. The international HCM Share registry showed that patients with a high body mass index have a significantly increased risk of heart failure, more advanced left ventricular outflow tract obstruction and more arrhythmias (i.e. HCM-related outcomes) [12]. Moreover, a prospective study in adolescent men demonstrated that even mildly elevated body weight in late adolescence significantly increased the risk to develop dilated cardiomyopathy in adulthood [13]. At the heart level, a recent proteomics study in human HCM tissue samples showed reduced levels of energy metabolism proteins [14]. This observation is in line with studies in human HCM showing energy deficiency of the heart [15, 16]. Energy deficiency has been proposed as the primary variant-induced pathomechanism of HCM [17], which is supported by studies showing reduced cardiac efficiency in preclinical asymptomatic carriers of sarcomere gene variants in the absence of cardiac hypertrophy [16, 18]. Accordingly, DCM caused by *TTN *gene variants has been linked with mitochondrial dysfunction and metabolic perturbations as cause of disease progression [19]. Overall, these studies indicate that timely disease stage-specific treatment of metabolic perturbations may slow down disease progression in cardiomyopathy patients [20]. An observational cohort to determine the predictive value of metabolic biomarkers (BIO FOr CARe: identification of biomarkers for development and progression of HCM in carriers of the Dutch *MYBPC3* founder carriers) and a clinical trial using metabolic drug therapy aimed to improve energetics of the heart at preclinical stage in HCM gene variant carriers are currently being performed by several DOSIS principal investigators (ENERGY trial) [21].

## Altered protein quality control as disease modifier in cardiomyopathy

An age-related decline in protein quality control (PQC) has been proposed as a contributor to disease progression in cardiomyopathy. As sarcomere proteins are the most abundant proteins in the heart, maintenance of sarcomere structure and function depends on PQC mechanisms. Pathogenic gene variants result in poison polypeptides or reduced protein levels (haploinsufficiency) and may trigger PQC and/or stress cellular protein homeostasis. DCM patients with truncating *TTN *variants show a relatively mild disease course, though with significant excess mortality in elderly patients. The latter may be explained by an age-related deterioration of the PQC mechanisms. As life expectancy increases, *TTN*-associated morbidity and mortality will likely become more prevalent [22]. Also in PLN-associated cardiomyopathy, protein aggregation and activation of PQC pathways has been observed in end-stage disease [23].

Terminally misfolded and aggregation-prone proteins are cleared by the two degradation systems, the ubiquitin-proteasome system and autophagy [24]. Furthermore, pathways of PQC are strongly linked to cell architecture, such as the microtubules network. DOSIS studies in a large set of cardiac tissues from a well-characterised HCM patient group showed altered PQC with several specific changes in gene-variant positive patients (genotype-positive) compared with genotype-negative patients and non-failing controls [25]. Heat shock proteins (HSP) involved in protein stabilisation (HSPB1) and refolding (HSPD1, HSPA2) were increased in genotype-positive HCM compared with controls. In addition, tubulin and acetylated-tubulin levels were significantly higher in HCM compared with controls, especially in HCM with truncating variants in *MYBPC3*, which cause protein haploinsufficiency. cMyBP‑C protein levels were inversely correlated with α‑tubulin levels suggesting a compensatory tubulin response to maintain cardiomyocyte structure, though this may be at the expense of cardiac function. Our study indicates that proliferation of the microtubular network represents a novel pathomechanism in cMyBP‑C haploinsufficiency-mediated HCM. Recent studies in human heart failure identified a central role for detyrosinated microtubules in regulating cardiomyocyte function and demonstrated the functional benefit upon reversal of this modification [26, 27]. This is of clinical importance since it represents a potential treatment target to improve cardiac function in HCM.

## Cell-to-cell mRNA/protein variability as pathomechanism in cardiomyopathy

As familial cardiomyopathies represent an autosomal dominant genetic disorder, most patients are heterozygous for the mutation and carry one variant and one normal wild-type allele. In cardiomyopathy patients, the heart of a genotype-positive individual produces the variant protein in addition to the normal protein. As indicated above, the homeostasis of cellular proteins is tightly regulated by the PQC system, but it is also regulated at the mRNA level by non-sense mediated mRNA decay. Both systems are needed to suppress the accumulation of variant protein while keeping the normal protein at sufficient levels in cardiac muscle cells. It was recently shown that transcription of both alleles occurs independently and in a stochastic manner, where one cell favours one allele and the next cell favours the other allele [28]. This burst-like, stochastic on/off switching of allele transcription does not affect mRNA and protein levels in case of homozygous wild-type alleles. However, heterozygosity of alleles as present in genotype-positive individuals may introduce cell-to-cell variation with one cardiomyocyte expressing high levels of variant protein, while variant levels may be low in another cardiomyocyte [29]. Indeed, it has been shown that *MYH7* gene variants cause a variable variant to wild-type ratio of mRNA expression in cardiomyocytes from the same heart [28, 30]. The DOSIS consortium showed intercellular variation of cMyBP‑C myofilament protein expression due to truncating *MYBPC3* variants in the myocardium of HCM patients [31]. The functional consequences of the variable protein expression, which results in a mosaic pattern of cardiomyocytes with low and high variant/wild-type expression, remain to be determined. Loss of cMyBP‑C causes severe dysfunction in mouse studies and engineered heart tissue [32, 33]. We propose that the intercellular variation of cMyBP‑C protein levels causes inhomogeneous contraction and relaxation and underlies the formation of myofibrillar disarray, a currently unexplained disease characteristic of HCM. As ageing reduces the quality of PQC, an age-dependent progression of the degree of allelic imbalance and cell-to-cell variation may contribute to cardiomyopathy development.

In conclusion, monogenetic cardiomyopathies have been intensely studied in the last three decades, and this has resulted in major progress in understanding what genes are involved. On the other hand, the striking heterogeneity, the highly variable age of onset, and the presence of gene variant carriers that never develop disease is as of yet largely unexplained. Given the profound repercussion for carriers, patients and family members we must improve our understanding of the individual’s response to the presence of a pathogenic gene variant.

**Fig. 3:**
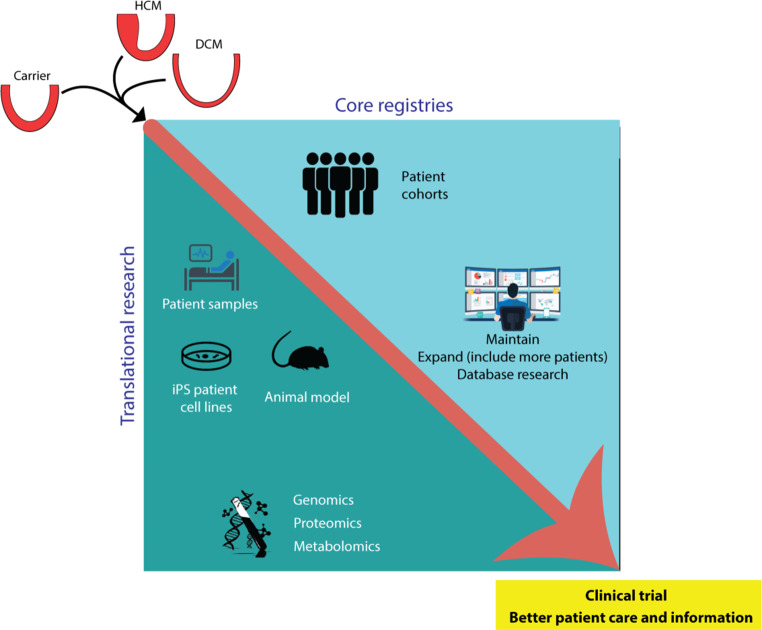
By combining cellular, genetic and clinical data from well-phenotyped national patient cohorts, DOSIS strives to define disease factors that in addition to the pathogenic gene variant cause and aggravate cardiac disease in cardiomyopathy patients

DOSIS aims to study unexplored mechanisms that will probably modify the pathogenic gene variant (Fig. 3). We have set up important initiatives and collaborations and have generated preliminary results showing that environmental and genetic modifiers are indeed important in our understanding. In the future we will step up our initiatives and projects and have identified an agenda, which contains—what we feel—important additional factors that when fully understood will guide clinicians in proper diagnosis, risk prediction, prognostication and, ultimately, cause-specific novel treatments.
